# Investigation of the cytotoxicity induced by cannabinoids on human ovarian carcinoma cells

**DOI:** 10.1002/prp2.1152

**Published:** 2023-12-15

**Authors:** Kartheek Sooda, Simon J. Allison, Farideh A. Javid

**Affiliations:** ^1^ Department of Pharmacy, School of Applied Sciences University of Huddersfield Huddersfield UK; ^2^ Department of Biological & Geographical Sciences, School of Applied Sciences University of Huddersfield Huddersfield UK

**Keywords:** apoptosis, cannabinoids, CBD, CBG, ovarian cancer

## Abstract

Cannabinoids have been shown to induce anti‐tumor activity in a variety of carcinoma cells such as breast, prostate, and brain. The aim of the present study is to investigate the anti‐tumor activity of cannabinoids, CBD (cannbidiol), and CBG (cannabigerol) in ovarian carcinoma cells sensitive and resistant to chemotherapeutic drugs. Sensitive A2780 cells and resistant A2780/CP70 carcinoma cells and non‐carcinoma cells were exposed to varying concentrations of CBD, CBG, carboplatin or CB_1_ and CB_2_ receptor antagonists, AM251 and AM630, respectively, alone or in combination, at different exposure times and cytotoxicity was measured by MTT assay. The mechanism of action of CBD and CB in inducing cytotoxicity was investigated involving a variety of apoptotic and cell cycle assays. Treatment with CBD and CBG selectively, dose and time dependently reduced cell viability and induced apoptosis. The effect of CBD was stronger than CBG in all cell lines tested. Both CBD and CBG induced stronger cytotoxicity than afforded by carboplatin in resistant cells. The cytotoxicity induced by CBD was not CB_1_ or CB_2_ receptor dependent in both carcinoma cells, however, CBG‐induced cytotoxicity may involve CB_1_ receptor activity in cisplatin‐resistant carcinoma cells. A synergistic effect was observed when cannabinoids at sublethal doses were combined with carboplatin in both carcinoma cells. The apoptotic event may involve loss of mitochondrial membrane potential, Annexin V, caspase 3/7, ROS activities, and cell cycle arrest. Further studies are required to investigate whether these results are translatable in the clinic. Combination therapies with conventional cancer treatments using cannabinoids are suggested.

## Graphical abstract

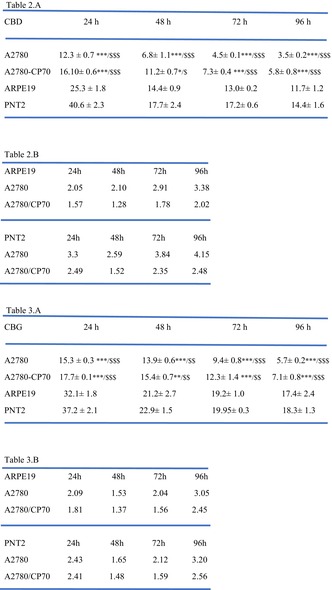

## 1 INTRODUCTION

Ovarian cancer is the deadliest among gynecological cancers. There are approximately 7500 new cases every year and approximately 35% of women diagnosed with ovarian cancer survive for 10 years.^1^ The majority of cases are diagnosed at stage 4. The rate of relapse is high and is around 2 years. The treatment with chemotherapeutics after relapse is much more intense, which leads to more toxicity to healthy organs, adverse effects, increase in drug resistance, and poor quality of life for the patients.^2^, ^3^ At the early stage of the disease, surgery and in advanced stages both surgical resection and systemic chemotherapy are standards of care.^4^, ^5^, ^6^, ^7^, ^8^, ^9^ In 2017, the FDA approved Olaparib in high‐grade serous ovarian cancer cells independent of BRACA mutations for recurrent ovarian cancer.^3^, ^10^ Bevacizumab was approved in 2018, which targets vascular endothelial growth factor, along with carboplatin and paclitaxel for stage III/IV relapsed cancer patients with platinum resistance.^11^, ^12^ However, the prognosis remains poor and due to multiple drug resistance in some patients.

Cannabinoids have been shown to inhibit the growth of tumor cells in culture and animal models by modulating key cell signaling pathways when tested on variety of cancer cell lines including lung, glioma, pancreas, lymphoma, lung, skin, prostate, colon, uterus, and breast carcinoma cells.^13^, ^14^, ^15^, ^16^, ^17^, ^18^, ^19^ It has been reported that the endocannabinoid system is expressed in the female reproductive tissues such as the ovaries.^20^ The endocannabinoid system consists of the ligands such as anandamide (AEA) and 2‐arachidonylglycerol (2‐AG), palmitoylethanolamide (PEA), receptors such as CB_1_, CB_2_, and GPR55 receptors, and the enzymes monoacylglycerol lipase (MAGL) and fatty acid amide hydrolase (FAAH).^15^, ^21^ Both CB_1_ and CB_2_ receptors are expressed in ovaries and their level of expression increases at the time of ovulation.^20^ There are data that suggested that the levels of AEA, PEA, and OEA (oleoylethanolamide, which is a bioactive endogenous ethanolamide fatty acid which is very similar in structure to the endocannabinoid ligands) increased in the follicular fluids in patients diagnosed with ovarian cancer and those with ovarian cysts.^22^ The level of expression of CB_1_ receptors was also reported to change depending on the stage and nature of the cancer, with low levels in benign and borderline epithelial rat ovarian tumors and high levels in invasive tumors.^23^ High level of FAAH expression was also reported in ovarian cancer patients.^24^ In addition, the levels of lysophospholipids were elevated in the blood and ascitic fluids in ovarian cancer patients.^25^ The accumulated studies suggest that endocannabinoids play a pivotal role in the healthy status of ovaries and also pathological conditions such as cancers. Therefore, endocannabinoid system is a therapeutic target for further research.

While the presence of psychoactive effects of cannabinoids such as THC prevented any progress in this field, it became apparent that cannabinoids such as CBG (cannabigerol) and CBD (cannabidiol) possess antiproliferative and cytotoxicity properties. CBD has been reported to induce cytotoxicity in various human carcinoma cells.^26^ CBD has low affinity for CB_1_ and CB_2_ receptors, acts as an antagonist or a non‐competitive negative allosteric modulator toward CB_1_ receptors, and as an inverse agonist toward CB_2_ receptors.^27^ CBD has been shown to have multiple molecular targets such as the regulation of Ca^2+^ levels, induction of ROS, regulating ATP, and proton leak suggest that the involvement of mitochondria is a CBD cellular mechanism.^15^, ^28^

CBG has a weak affinity for cannabinoid CB_1_ and CB_2_ receptors and was suggested to exert its mechanism of action through TRP receptor channels.^29^

The lack of a comprehensive study on the underlying mechanism of action of cannabinoids together with little knowledge on the effect of the combination of cannabinoids with chemotherapeutic drugs has hindered the development of cannabinoids as therapeutic agents to treat gynecological tumors. Therefore, the present study was designed to address this gap in our knowledge.

## 2 MATERIALS AND METHODS

### 2.1 Cell culture

Human ovarian carcinoma cells, A2780 (sensitive to chemotherapeutics), A2780/CP70 (resistant to chemotherapeutics), and non‐cancerous cells including PNT2 (normal prostate epithelial cell line), and/or ARPE19 (human retinal pigment cell line) were maintained according to the ATCC and ECACC guidelines. A2780 cell line has commonly been used and was established from an ovarian carcinoma cell from untreated patient. The cell line is sensitive to cancer chemotherapeutic drugs such as cisplatin and carboplatin that are used in the clinic.^30^ On the other hand, A2780/CP70 cells are resistant ovarian carcinoma cells which have been developed by repeated exposure to cisplatin.^31^

The cells were grown in complete media, RPMI and maintained with 5% CO_2_ at 37°C under humidified conditions. The cells were sub‐cultured when they reached 70% confluence. All compounds were dissolved in ethanol or filtered sterile water, and the final concentration of ethanol did not exceed 1%. Preliminary experiments revealed that 1% ethanol did not induce any change in the cells compared to media treated cells.

### 2.2 MTT assay

In vitro chemosensitivity assays were performed using the MTT assay as previously described.^32^, ^33^ Briefly, at 70% confluency, cells were seeded in 96‐well plates at a density of 2000 cells/well. Twenty‐four hours later, cells were exposed for 24, 48, 72, or 96 h to a range of concentrations of freshly prepared compounds (1 nM – 100 μM) or vehicle. After the allocated contact time had elapsed, the media was removed and 0.5% (w/v) MTT solution diluted in complete media (200 μL) was added to each well. Following a 4 h incubation, the supernatants were removed, and the formazan crystals were dissolved in dimethylsulfoxide (150 μL). The absorbance was read at 540 nm on a Tecan Infinite 50 plate UV reader.

In separate experiments, cells were seeded in 96‐well plates and after 24 h elapsed they were exposed to different concentrations of drugs or vehicle. Cells were then left in the incubator for a further 24 or 48 h. Then the media was replaced with fresh media containing no drug and the cells were left in the incubator. MTT assay was performed after 96 h elapsed from the time when cells were exposed to drugs or vehicle.

Additionally, some experiments were carried out where cells were seeded in 96‐well plates and after 24 h elapsed they were exposed to CBD or CBG prior to the addition of a second drug such as carboplatin or cannabinoid antagonists such as AM251 and AM630. The cells were then left in the incubator and MTT assay was performed 96 h following the initial exposure to the drugs.

### 2.3 Selectivity Index and Combination Index

Preferential cytotoxicity of CBD and CBG toward cancer cells was determined by calculating the selective index. The selective index (SI) is the ratio of mean IC_50_ values for non‐cancer cells to cancer cells. If SI >1, this means the drug shows preferential cytotoxicity toward cancer cells, if SI = 1 it means that the drug is equally toxic to both cancer and non‐cancer cells. If SI <1, this means that the drug is more toxic to non‐cancer cells than cancer cells.^33^

While the isobologram can be considered predominantly as a graphical depiction of drug interactions, the combination index (CI) is a quantitative representation. It relates the actual drug concentration in the combination to the concentration of the single drug that achieves the same effect. Other groups^34^ have also used CI to interpret the data when drugs were used in combinations. Here, CI analysis was used to investigate combinations at 50% cytotoxicity. To this end the IC50 of the viability dose–response curves were determined by non‐linear regression curve fit analysis (GraphPad Prism, v5.00).

The CI for drug A (CBD or CBG), when they were combined with drug B (carboplatin) was calculated by the ratio of the applied concentration of drug A (100 nM) in combination to the IC_50_ of A when it was applied individually, the ratio of the applied concentration of drug B in combination (combination IC_50_ – concentration of drug A), to IC_50_ of B on the cells^34^, ^35^:

$${\mathrm{CI}}_{A+B}=\left(C_{A}÷{\mathrm{IC}}_{50}\;A\right)+\left(C_{B}÷{\mathrm{IC}}_{50}\;B\right)$$

CI_A+B_ is the combination index of A and B; *C*_A_ the concentration of A applied in combination; *C*_B_ the concentration of B applied in combination; IC_50_ A the IC_50_ of drug A when it was applied individually to the cells; IC_50_ B = IC_50_ of drug B when it was applied individually to the cells.

The calculated CIs were compared with those in Table 1.

**TABLE 1:** Indication of combination effects relative to combination index values. Adopted from Bijnsdorp et al.^34^

CI value	Combination effect
>0.1	Very strong synergism
0.1–0.3	Strong synergism
0.3–0.7	Synergism
0.7–0.85	Moderate synergism
0.85–0.9	Slight synergism
0.9–1.1	Nearly additive
1.1–1.2	Slight antagonism
1.2–1.45	Moderate antagonism
1.45–3.3	Antagonism
3.3–10	Strong antagonism

### 2.4 Mitochondrial membrane potential assay

The mitochondrial membrane potential assay was performed to detect the loss in mitochondrial potential (de‐polarization), which is a vital step in apoptosis.^36^ Cells were seeded in T25 flasks at 4 × 10^5^ cells per flask and were incubated for 48 h or until they reached confluency. After the incubation time elapsed, drugs at concentrations of 10, 30, and 50 μM or vehicle were added to the flasks and further incubated for 24 h. After 24 h elapsed, the cells were harvested and suspended in PBS. Cell viability and number were determined by the cell viability assay using via1 cassettes on NC‐3000 system. The samples were diluted in PBS to 1 × 10^6^ cells/mL and 2.5 μg/mL of JC‐1 dye was added to each sample. JC‐1 is a negatively charged dye, which stains as fluorescent red as it aggregates in the mitochondrial matrix. In the absence of mitochondrial membrane potential, JC‐1 localizes in the cytosol in its fluorescent green form. Following the addition of JC‐1, the cells were incubated for 10–30 min at 37°C. After incubation, cells were washed twice with 1× PBS without disturbing the pellet, and the pellet was resuspended in 0.25 mL of 1 μg/mL DAPI. DAPI stains as florescent blue in late apoptotic and necrotic cells by binding to DNA. 12.5 μL of samples were loaded on to an A‐8 slide and were analyzed using the NC‐3000 image cytometry. The degree of apoptosis (in proportional to the loss in mitochondrial membrane potential) was measured using a scatter plot of JC‐1 red fluorescence versus JC‐1 green fluorescence.

### 2.5 Annexin V assay

The Annexin V assay was performed to differentiate healthy cells from early and late apoptotic cells. Cells were seeded in T25 flasks at 2.75 × 10^5^ cells per flask and were incubated for 24 h. After the incubation time elapsed, drugs at concentrations of 10, 30, and 50 μM or vehicle were added to the flasks and further incubated for 24 h. Cells were suspended in PBS at a density of 4 × 10^5^ cells/mL, followed by centrifugation at 400 *g* for 5 min. The supernatant was removed and replaced with 100 μL of Annexin V mix, 94 μL of Roche buffer, 2 μL of Annexin V‐CF488A, 2 μL of 10 μg/mL of propodium iodide (PI), and 2 μL of Hoechst 33342 buffer. Hoechst 33342 buffer in the Annexin V mix binds to the nuclei in the sample and fluoresces violet. The early apoptotic cells are stained with Annexin V‐CF488A, which fluoresces green. The non‐viable cells are stained by PI, and the late apoptotic cells are stained by both PI and Annexin V‐CF488A, which fluoresces red and green, respectively. Following the addition of Annexin V mix, samples were incubated at 37°C for 20 min in the dark with regular mixing to keep the cells in suspension. After the incubation time elapsed, 40 μL of samples were loaded on to an A‐2 slide and were analyzed immediately using the NC‐3000 system. The scatter plot of the fluorescence intensity of Annexin V‐CF488A versus the fluorescence intensity of PI was used as a representation of cells in different stages of apoptosis.

### 2.6 Caspase 3/7 assays

Caspase 3/7 assays were carried out to determine the involvement of caspases in the execution of apoptosis induced by CBD and CBG on ovarian cancer cells.

#### 2.6.1 Sensolyte homogenous caspase 3/7 assay

Sensolyte homogenous caspase 3/7 assay kit was used to determine the caspase activity in ovarian cancer cells when treated with CBG. Cells were seeded in 96‐well plate at 6 × 10^3^ cells per well and were incubated for 24 h. CBG was added in concentrations of 10, 20, 30, and 50 μM and incubated for a further 24 h. After the incubation time elapsed, the media was removed and replaced with 150 μL/well of fresh complete media. Caspase 3/7 solution was prepared by diluting the caspase substrate 1:100 with DTT‐containing assay buffer. 50 μL/well caspase substrate was added to the plates, and the cells were incubated for 30 min. Ac‐DEVD‐AMC serves as the fluorogenic indicator for assaying caspase‐3/7 activity. Ac‐DEVD‐AMC generates the AMC fluorophore in the presence of caspase 3/7, which produces bright blue fluorescence. The fluorescence was measured by the FLOUstar OPTIMA plate reader with the excitation at 354 nm and emission at 442 nm.

#### 2.6.2 ApoTox‐Glo™ trip assay

The level of caspase 3/7 was also determined using Apotox‐Glo Triple assay. Cells were seeded and drugs were added as described above in Section 2.6.1. After 24 h incubation, caspase 3/7 reagent was prepared by mixing caspase buffer and substrate and 100 μL of reagent was added to each well for 30 min incubation at room temperature. The pro‐luminescent substrate DEVD‐Aminoluciferin will be activated in the presence of caspase3/7 enzyme. The luminescence was measured by the FLOUstar OPTIMA plate reader.

### 2.7 Cell cycle analysis

Cells were seeded in T25 flasks at 4 × 10^5^ cells per flask and were incubated for 48 h or until they reached confluency. Freshly prepared drugs at concentrations of 10, 30, and 50 μM or vehicle were added to the flasks and further incubated for 24 h. After the incubation time elapsed cells were suspended in PBS with a density closer to 1 × 10^6^cells/mL. The cells were washed twice with 1× PBS before the addition of 250 μL of lysis buffer (solution 10) supplemented with 10 μg/mL DAPI (solution 12) was added to the samples and incubated for 5 min at 37°C. After incubation, 250 μL of stabilization buffer (solution 11) was added. 12.5 μL of samples were loaded on to an A‐8 slide and were analyzed using the NC‐3000 system.

### 2.8 Data analysis

Data were presented as the mean ± SE of the mean. At least three independent experiments were carried out to determine the IC_50_ (*n* ≥ 3). Statistical analyses were carried out using Graph pad 7, one‐way ANOVA with Dennett's multi‐comparison test. A value of *p* < .05 was considered statistically significant compared to control values.

### 2.9 Materials

CBD, CBG, carboplatin, cannabinoid antagonists, AM251, and AM630 were purchased from Tocris, UK. Phosphate‐buffered saline (PBS), Solution 8 (1 μg/mL 4′,6‐diamidino‐2‐phenylindole [DAPI]), Solution 7 (5,5,6,6‐tetrachloro‐1,1,3,3‐tetraethylbenzimidazolcarbocyanine iodide of 200 μg/mL JC‐1), Annexin V binding buffer (10× concentrate), Solution 16 (500 μg/mL Propidium Iodide), Solution 10 (Lysis buffer), Solution 11 (stabilization buffer), Solution 12 (500 μg/mL DAPI), NC‐Slide A8™, NC‐Slide A2™ glass slides and via‐1 cassettes were purchased from ChemoMetec, Denmark. NC‐3000™ image cytometer was used to perform the assays. Apotox‐Glo Triple assay was purchased from Promega, UK. Sensolyte homogenous caspase 3/7 assay kit was purchased from Anaspec, UK. Solution 15, Hoechst 33342 buffer was purchased from Thermo‐Scientific, UK. Roche buffer was purchased from BD Bioscience, UK, Annexin V‐CF488A was purchased from Santa Cruz Biotechnology, UK. MTT, DMSO, and media for growing cell lines and all supplements were purchased from Sigma Aldrich, UK and Life Technologies, UK, and cell lines were purchased from ATCC.

## 3 RESULTS

### 3.1 Cytotoxicity induced by cannabinoids

**FIGURE 1:**
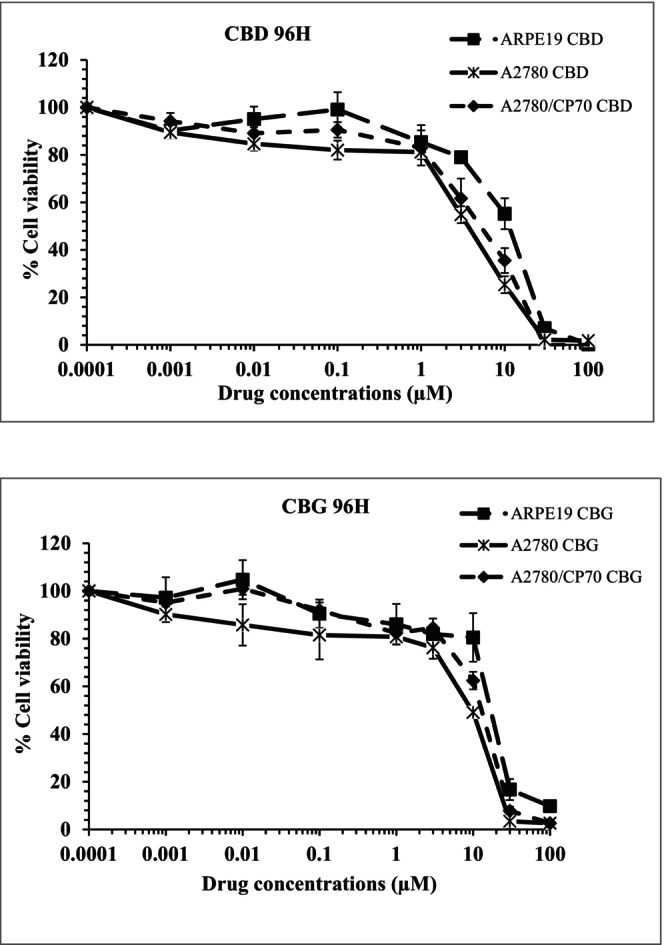
Representative data indicating the effect of CBD or CBG (1 nM to 100 μM) on ovarian cancer cells A2780, A2780/CP70 and non‐cancer cells, ARPE19 (retinal epithelial cells), over 96 h contact times. Data represent the mean ± SE of *n* = 4.

The application of CBD or CBG (1 nM–100 μM) induced dose‐ and time‐dependent cytotoxicity which were significant (*p* < .05, .01, .001) at concentrations higher than 1 μM when tested on all cell lines compared to the vehicle‐treated cells (Figure 1). CBD was more potent than CBG in inducing cytotoxicity in A2780 cells compared to cisplatin‐resistant A2780/CP70 cells at all time points tested.

The cytotoxicity afforded by both CBD and CBG increased with increasing exposure contact time in all cell lines tested. The IC_50_ values for CBD and CBG in carcinoma cells were significantly (*p* < .05, .01, and .001) lower than those in non‐carcinoma cells (Tables 2 and 3). For example, the IC_50_ values for CBD in A2780 cells were 12.3 ± 0.7 μM, 6.8 ± 1.07 μM, 4.5 ± 0.2 μM, and 3.5 ± 0.2 μM following 24, 48, 72, and 96 h contact times which were lower when compared to those in non‐carcinoma cells, ARPE19 and PNT2 cells (Table 2).

**TABLE 2:** (A) IC_50_ values (μM) for CBD‐induced cytotoxicity on ovarian cancer A2780, A2780/CP70 cells, and non‐cancer ARPE19 and PNT2 cells over different contact times. (B) Selectivity index of CBD‐induced toxicity as compared to ARPE19 and PNT2 cells.

(A)				
CBD	24 h	48 h	72 h	96 h
A2780	12.3 ± 0.7^***,$$$^	6.8 ± 1.1^***,$$$^	4.5 ± 0.1^***,$$$^	3.5 ± 0.2^***,$$$^
A2780/CP70	16.10 ± 0.6^***,$$$^	11.2 ± 0.7^*,$^	7.3 ± 0.4^***,$$$^	5.8 ± 0.8^***,$$$^
ARPE19	25.3 ± 1.8	14.4 ± 0.9	13.0 ± 0.	11.7 ± 1.2
PNT2	40.6 ± 2.3	17.7 ± 2.4	17.2 ± 0.6	14.4 ± 1.6

(B)				
ARPE19	24 h	48 h	72 h	96 h
A2780	2.05	2.10	2.91	3.38
A2780/CP70	1.57	1.28	1.78	2.02
PNT2	24 h	48 h	72 h	96 h
A2780	3.3	2.59	3.84	4.15
A2780/CP70	2.49	1.52	2.35	2.48

*Note*: *p < .05 and ***p < .001 compared to AREPE19, and ^$^ *p* < .05, ^$$$^ *p* < .001 compared to PNT2 cells.

**TABLE 3:** (A) IC_50_ values (μM) for CBG‐induced cytotoxicity on ovarian cancer A2780, A2780/CP70 cells and non‐cancer ARPE19 and PNT2 cells over different contact times. (B) Selectivity index of CBG‐induced toxicity as compared to ARPE19 and PNT2 cells.

(A)				
CBG	24 h	48 h	72 h	96 h
A2780	15.3 ± 0.3 ***/$$$	13.9 ± 0.6^***,$$^	9.4 ± 0.8^***,$$$^	5.7 ± 0.2^***/$$$^
A2780/CP70	17.7 ± 0.1^***,$$$^	15.4 ± 0.7^**,$$^	12.3 ± 1.4^***,$$^	7.1 ± 0.8^***,$$$^
ARPE19	32.1 ± 1.8	21.2 ± 2.7	19.2 ± 1.0	17.4 ± 2.4
PNT2	37.2 ± 2.1	22.9 ± 1.5	19.95 ± 0.3	18.3 ± 1.3

(B)				
ARPE19	24 h	48 h	72 h	96 h
A2780	2.09	1.53	2.04	3.05
A2780/CP70	1.81	1.37	1.56	2.45
PNT2	24 h	48 h	72 h	96 h
A2780	2.43	1.65	2.12	3.20
A2780/CP70	2.41	1.48	1.59	2.56

*Note*: **p < .01 and ***p < .001 compared to AREPE19, and ^$$^ *p* < .01, ^$$$^ *p* < .001 compared to PNT2 cells.

The IC_50_ values for CBG when tested on A2780 carcinoma cells were also significantly (*p* < .01 and <.001) lower, 15.3 ± 0.3 μM, 13.9 ± 0.6 μM, 9.4 ± 0.8 μM, and 5.7 ± 0.2 μM following 24, 48, 72, and 96 h contact times when compared to those in non‐carcinoma cells (Table 3).

The IC_50_ values for both CBD and CBG in A2780/CP70 cells were slightly higher than those observed in A2780 cells, although they were significantly (*p* < .001) lower than those in non‐carcinoma cells (Tables 2 and 3). The higher SI values for CBD indicated the selectivity of CBD‐induced cytotoxicity in carcinoma cells than in non‐carcinoma cells.

**FIGURE 2:**
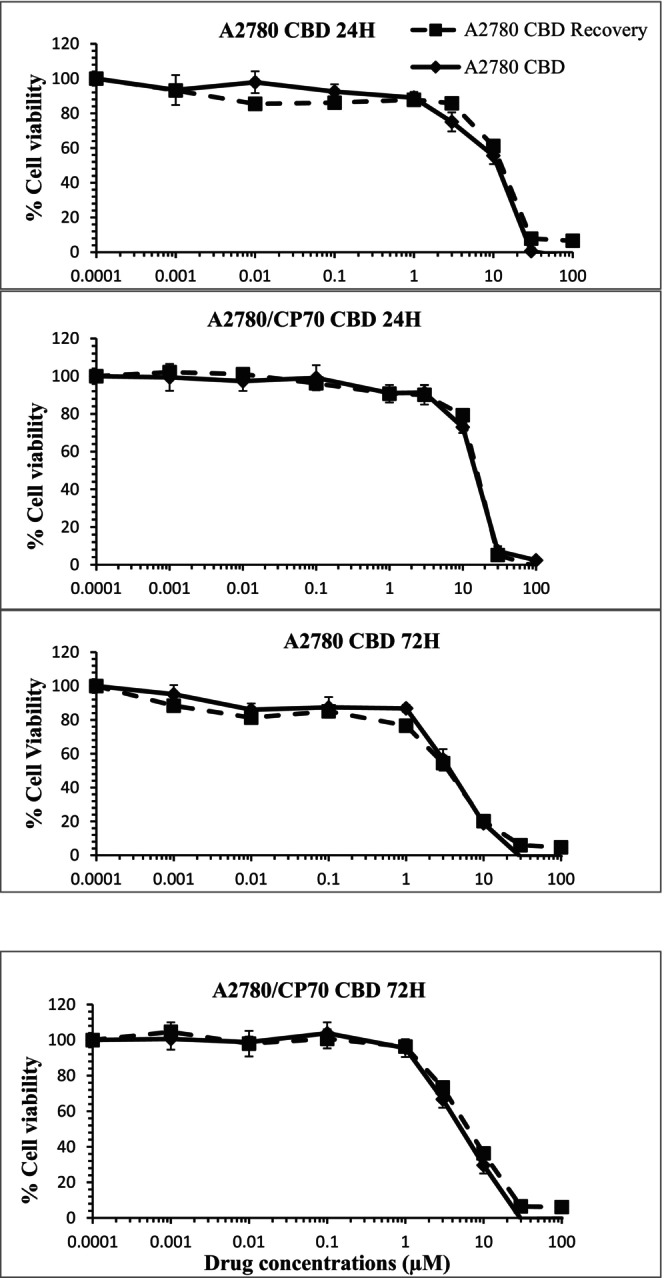
Representative data indicating the concentration response curves to CBD when cells were allowed to recover from exposure to CBD compared to when cells were in continuous contact with cannabinoids prior to the MTT assay over different contact times. Data represent the mean ± SE of *n* = 4.

In separate experiments, attempts were made to investigate if the cytotoxicity induced by cannabinoids can be overcome by withdrawing the drug. In other words, it was tested whether carcinoma cells can recover after the initial exposure to cannabinoids. The cells were treated with CBD or CBG for 24, 48, and 72 h and then were washed out of the drugs and fresh media was added to the cells (without cannabinoids). The MTT assay was carried out at 96 h from the initial addition of cannabinoids. No significant differences were observed in CBD‐ and CBG‐induced cytotoxicity when drugs were withdrawn compared to the experiments where cannabinoids were in continuous contact with the cells prior to the MTT assay at all time points tested (Figure 2). The results showed that cannabinoids exert irreversible cytotoxicity in the carcinoma cells.

### 3.2 Cytotoxicity induced by carboplatin alone and in combination with cannabinoids

Carboplatin when used alone did not induce significant cytotoxicity after 24 h exposure time. However, further contact time induced cytotoxicity with measurable IC_50_s. The IC_50_ values have been summarized in Table 4. Carboplatin induced much less cytotoxicity in A2780/CP70 cells compared to A2780 cells in all contact points tested. Notably, when cells were treated for 96 h, the IC_50_s were 2.57 ± 0.3 μM and 44.9 ± 3.5 μM in A2780 and A2780/CP70 cells, respectively. In separate experiments, recovery tests were carried out when cells were washed out of carboplatin after an initial exposure for 24, 48, and 72 h and then subjected to MTT. Such studies showed a greater IC_50_ for carboplatin at all contact points (Table 4).

**TABLE 4:** IC_50_ values (μM) for carboplatin‐induced cytotoxicity on ovarian cancer A2780, A2780/CP70 cells, and non‐cancer ARPE19 cells over different contact times.

Carboplatin	24 h	48 h	72 h	96 h
A2780	>100	17.3 ± 0.6	4.3 ± 0.7	2.6 ± 0.3
A2780/CP70	>100	>100	48.0 ± 1.9	44.9 ± 3.5
Carboplatin recovery	24 h	48 h	72 h	
A2780	7.9 ± 0.5	4.8 ± 0.2	2.6 ± 0.5	
A2780/CP70	66.5 ± 3.4	48.7 ± 0.4	45.5 ± 2.6	

**FIGURE 3:**
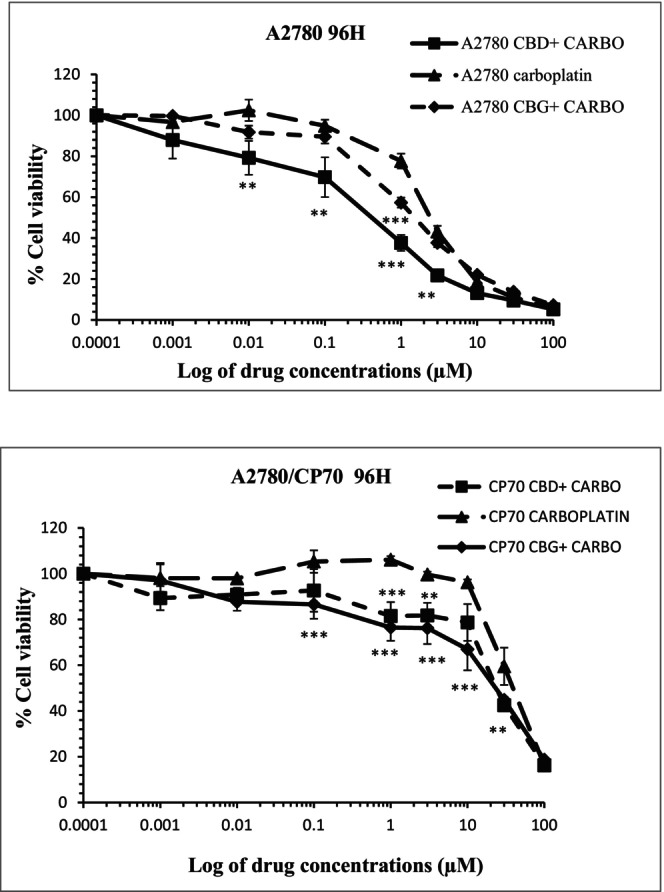
Representative data showing the effect of carbachol alone or in combination with CBD (100 nM) or CBG (100 nM) on A2780 and A2780/CP70 carcinoma cells over a contact time of 96 h. Data represent the mean ± SE of *n* = 4. **p < .01 and ***p < .001 compared to control values.

**FIGURE 4:**
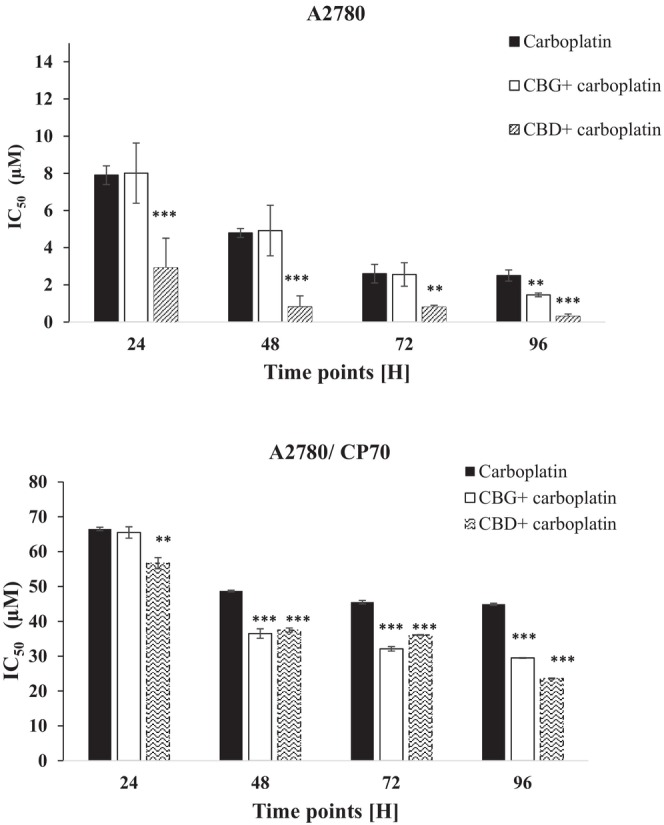
Carboplatin IC50 alone and in combination with cannabinoids in A2780 and A2780/CP70. Data represent the mean ± standard error of *n* = 4. **p < .01 and ***p < .001 compared to control values.

In separate experiments, cells were exposed to CBD or CBG at a sublethal concentration of 100 nM and after 30 min elapsed, they were treated with varying concentrations of carboplatin and further incubated for different contact times of 24, 48, and 72 h. After the allocated time elapsed, the cells were washed with fresh media (without carboplatin) and left for certain contact times up to 96 h prior to carrying out the MTT assay. Results showed that a combination of CBD plus carboplatin induced greater cytotoxicity when compared to the cells which were exposed to carboplatin alone at all time points tested (Figure 3). When cells were treated with a combination of CBD plus carboplatin, the IC50s were significantly (*p* < .001) reduced from 7.9 ± 0.5 μM, 4.7 ± 0.2 μM, 2.6 ± 0.5 μM, and 2.5 ± 0.3 μM to 2.91 ± 1.62 μM, 0.82 ± 0.62 μM, 0.82 ± 0.13 μM, and 0.23 ± 0.13 μM (when cells were treated with carboplatin alone), over 24, 48, 72, and 96 h, respectively (Figure 4). When CBG was added prior to the addition of carboplatin, the IC_50_ values were significantly (*p* < .01) reduced to 1.46 ± 0.5 μM only over a 96 h contact time (Figure 4). The CI values of carboplatin in combination with CBD were 0.36, 0.17, 0.15, and 0.096 following 24, 48, 72, and 96 h contact times, respectively, suggesting a strong synergism in A2780 cells. CBG also induced synergism when combined with carboplatin only over 96 h exposure time (CI = 0.55).

In A2780/CP70 carcinoma cells, carboplatin in combination with 100 nM CBD significantly (*p* < .001) showed lower IC_50_ values (56.7 ± 2.6 μM, 37.5 ± 1.2 μM, 36.1 ± 1.1 μM, and 23.6 ± 0.7 μM) than those afforded by carboplatin alone (66.5 ± 3.3 μM, 48.7 ± 0.4 μM, 45.6 ± 0.5 μM, and 44.9 ± 3.2 μM). In addition, carboplatin in combination with 100 nM CBG achieved significantly lower (*p* < .001) IC_50_ values of 36.5 ± 3.2 μM, 32.1 ± 2.7 μM, and 29.5 ± 0.5 μM at 48, 72, and 96 h, respectively. Furthermore, when A2780/CP70 cells were treated with a combination of carboplatin plus CBD, slight synergistic effects were observed with CI values of 0.85, 0.78, 0.8, and 0.57 after 24, 48, 72, and 96 h contact times, respectively. Carboplatin in combination with CBG exerted similar effects at 48 h (0.78), 72 h (0.71), and 96 h (0.65) contact times.

Cytotoxicity induced by a combination of cannabinoids plus carboplatin was comparable to the cytotoxicity induced by carboplatin alone in non‐carcinoma cells, ARPE19 cells.

### 3.3 Mitochondrial membrane potential assay

**FIGURE 5:**
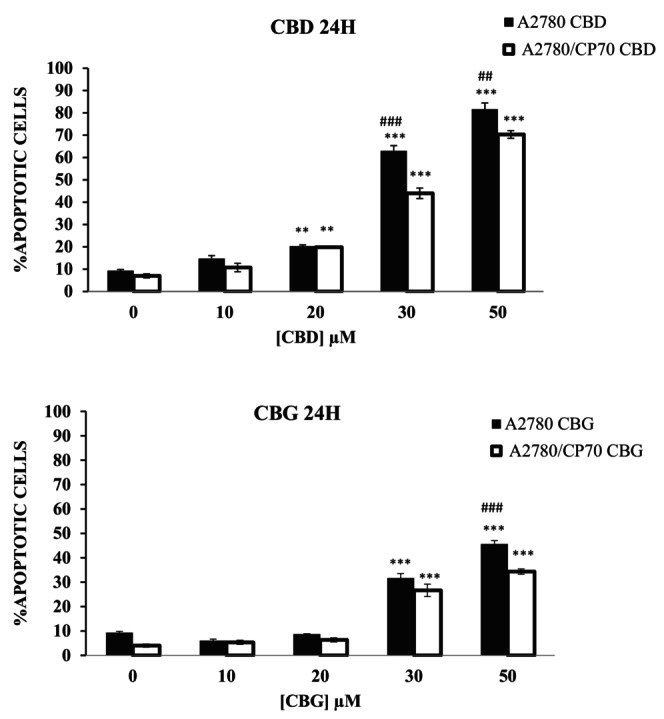
Mitochondrial membrane potential assay on A2780 cells treated with (a) CBD and (b) CBG. The percentage of apoptotic cells when treated with CBD at 10, 20, 30, and 50 μM on A2780 and A2780 CP70, respectively, after 24 h of contact time. Data represent the mean ± SE of the mean *n* = 3. **p < .01 and ***p < .001 relative to controls, and ^##^ *p* < .01, ^###^ *p* < .001 relative to the other cell line at the same concentration.

Figure 5 shows the effects of cannabinoids in inducing apoptotic cells in both A2780 and A2780/CP70 cells. Both CBD and CBG induced an increase in the percentage of apoptotic cells in a dose‐dependent manner in both cell lines examined (Figure 5). The results achieved significance (*p* < .01, <.001) at 20, 30, and 50 μM of CBD. Pre‐treatment with 30, and 50 μM CBD significantly (*p* < .001) induced greater percentage of apoptotic cells in A2780 cells as compared to cisplatin‐resistant A2780/CP70 cells. Significant results were also achieved (*p* < .01, <.001) when CBG was used at 30 and 50 μM. Pre‐treatment with CBG at 50 μM significantly (*p* < .001) induced a greater percentage of apoptotic cells in A2780 as compared to cisplatin‐resistant A2780/CP70 cells. The results correlated with greater cytotoxic effects of CBD and CBG on cisplatin‐sensitive cells compared to cisplatin‐resistant cells.

### 3.4 Annexin V assay

**FIGURE 6:**
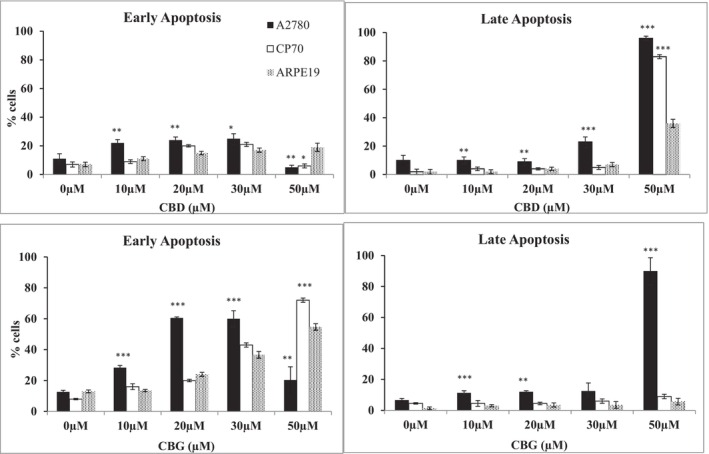
Representative data showing the percentage of cells in early apoptosis and late apoptosis when A2780, A2780 CP70, and ARPE19 were treated with 10, 20, 30, and 50 μM of CBD. Data represent the mean ± SE of the mean *n* = 3. *p < .05, **p < .01, and ***p < 0.001 relative to ARPE19 cells.

Pre‐treatment with CBD at 10, 20, and 30 μM induced early apoptosis in A2780 cells at 24 h contact time. However, at 50 μM of CBD, a higher percentage of cells were in the late apoptotic phase. Similar profile of action was observed in A2780/CP70 cells. There were significantly (*p* < .001) less percentage of apoptotic cells in both carcinoma cells compared to non‐cancer ARPE19 cells, which also correlated with the selective cytotoxicity of CBD toward ovarian cancer cells (Figure 6).

The percentage of apoptotic cells in A2780 cells were higher than those in A2780/CP70 and ARPE19 cells when treated with CBG for 24 h. There were 20 ± 6.4% and 90 ± 8.3% of cells in early apoptosis and late apoptosis in A2780 cells, respectively. The percentages of early and late apoptotic cells in A2780/CP70 were 72 ± 1.4% and 9 ± 1.4% compared to those in ARPE19 cells 54 ± 2.1% and 5.6 ± 1.8, respectively, when treated with 50 μM CBG. At 50 μM of CBG, both ovarian cancer cells significantly (*p* < .001) showed less percentage of cells in early apoptosis compared to non‐cancer ARPE19 cells. CBG also showed selective induction of apoptosis in the ovarian cancer cells tested compared to non‐ cancer ARPE19 cells.

There were more percentage of ovarian cancer cells in early apoptosis when treated with CBD as compared with those treated with CBG, which also correlates with the earlier results where CBD induced a stronger cytotoxicity compared to CBG. Furthermore, the results showed that the carcinoma cells were in early apoptosis when treated with 50 μM of CBG, whereas cells were in late apoptosis when treated with the same concentration, 50 μM of CBD. In addition, both cannabinoids induced a higher percentage of apoptotic cells in carcinoma cells than in non‐carcinoma cells.

### 3.5 Caspase 3/7 activity

**FIGURE 7:**
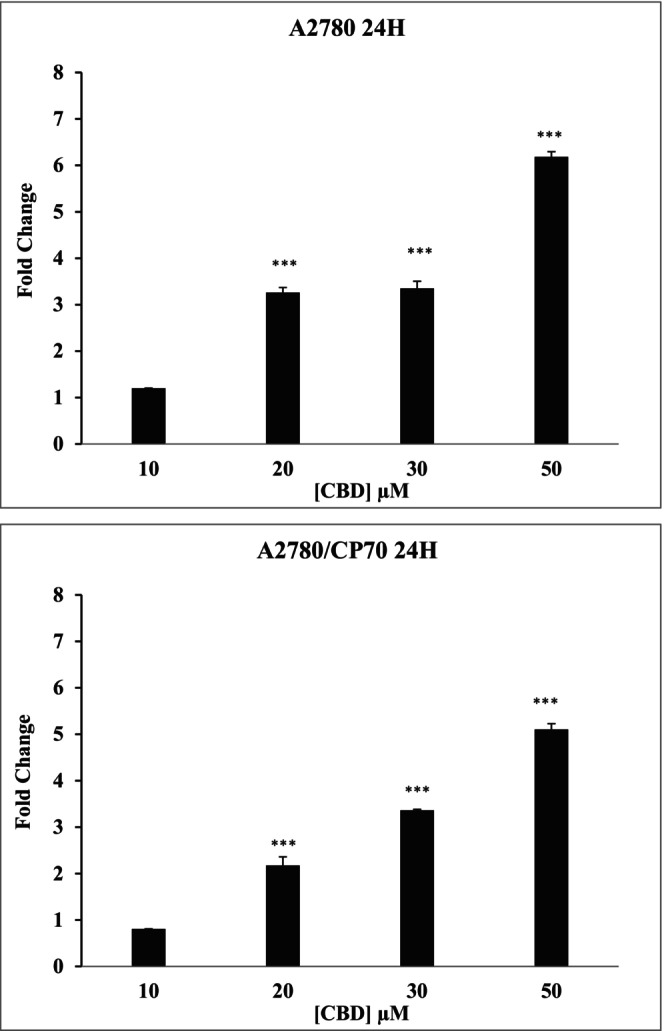
Representative data showing the fold change in caspase 3/7 activity of A2780 and A2780/CP70 when treated with CBD at different concentrations. Data represent mean ± SE of mean of *n* = 3. ***p < .001 compared to control values.

The protease activity of caspase 3/7 was quantified in ovarian cancer cells in the presence of CBD or CBG. Both CBD and CBG increased the caspase 3/7 activity in both carcinoma cells in dose‐dependent manner at 24 h contact time (Figure 7). The significance difference in the activity of caspases 3/7 was observed at concentrations higher than 10 μM in both cell lines treated with CBD or CBG.

### 3.6 Cell cycle analysis

The effects of CBD and CBG on the cell cycle were investigated by NC3000 image cytometry using DAPI, a fluorescent stain that binds to DNA. The change in DNA content at different stages of the cell cycle was observed by variation in DAPI fluorescence.

**FIGURE 8:**
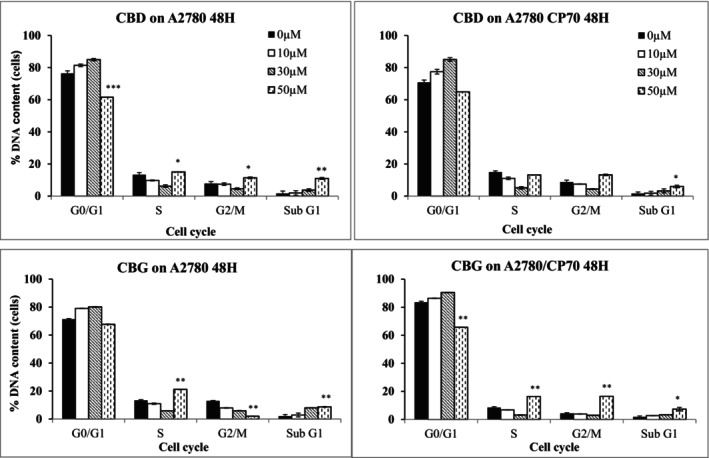
Different stages of the cell cycle when cells were exposed to CBD or CBG (10, 30, and 50 μM) for 48 h. Data showing the percentage of DNA content among CBD‐treated cells as a relative factor for different stages of the cell cycle compared to the control. Data represent the mean ± SE of mean of *n* = 3, *p < .05, **p < .01 and ***p < 0.001.

When A2780 cells were treated with CBD for 48 h, there was a slight increase in G0/G1 phase at 10 μM (81.4 ± 1.3%) and 30 μM CBD (85 ± 1.5%) compared to those in control cells (76.4 ± 2.5%; Figure 8). The increase in the G0/G1 phase correlated with a decrease in the S phase population of cells to 6 ± 1.5% compared to control cells, 13.3 ± 1.9 when cells were treated with CBD at 30 μM. The decrease in the G0/G1 at 50 μM CBD (61.6 ± 1.4%) was in line with a significant increase in the subG1 phase (*p* < .01) from control, 1.6 ± 0.4% to 11.1 ± 1.0% in the presence of 50 μM CBD. The latter observation suggested that 50 μM was too high concentration and exerted the cytotoxic effect on cells leading to DNA fragmentation, which further led to the induction of cell death.

Similar to CBD, when A2780 cells were treated with CBG, there was an increase in G0/G1 phase from control values of 71.3 ± 0.5% to 79.1 ± 0.4%, at 10 μM and 80.2 ± 1.3% at 30 μM. The increase in G0/G1 phase was correlated with a decrease in S phase compared to control values. The decrease in G0/G1 in A2780 cells treated with of 50 μM CBG (67.6 ± 1.1%) was in line with a significant increase in subG1 phase (*p* < .01) from control, 2 ± 1.2% to 8.7 ± 0.6%; the latter suggested DNA fragmentation when CBG was used at 50 μM. However, at 50 μM CBG, there was a significant increase (*p* < .01) in S phase and a significant decrease (*p* < .01) in G2/M phase compared to the control; this may suggest a growth arrest at S phase (Figure 8).

CBD exerted similar effects on cisplatin‐resistant A2780/CP70 cells compared to parental A2780 cells. The increase in G0/G1 phase from the control, 70.8 ± 1.4% to 77.4 ± 1.3% and 85.1 ± 1.1% when CBD was used at 10 and 30 μM, respectively, was followed by a decrease in the S phase. The decrease in G0/G1 phase when A2780 C70 cells were treated with 50 μM CBD (64.9 ± 0.9%) was explained by a significance increase (*p* < .01) in the subG1 phase in control cells from 1.5 ± 0.06% to 6 ± 0.9% (Figure 8).

After 48 h, there was an increase in G0/G1 from 83.6 ± 0.6% in control cells to 86.4 ± 0.5%, and 90.6 ± 0.4% when CBG was used at 10 and 30 μM, respectively, in A2780/CP70 cells. A decrease in S phase was observed at 30 μM CBG, 5.6 ± 0.6% from 13.3 ± 0.6% in control cells, which was followed by a significant increase when CBG was used at 50 μM. Similar to S phase, a significant increase (*p* < .01) in G2/M phase was observed at 50 μM CBG (16.4 ± 1.9%). The SubG1 phase increased (*p* < .01) from 1.9 ± 0.14% in control cells to 7.4 ± 0.6% when CBD was used at 50 μM suggesting a DNA fragmentation followed by cell death. The results demonstrated that CBG at the highest concentration of 50 μM CBG may induce growth arrest in S phase and G2/M phase in cisplatin‐resistant A2780/CP70 cells at 48 h time point.

### 3.7 Antioxidant activity of cannabinoids

**FIGURE 9:**
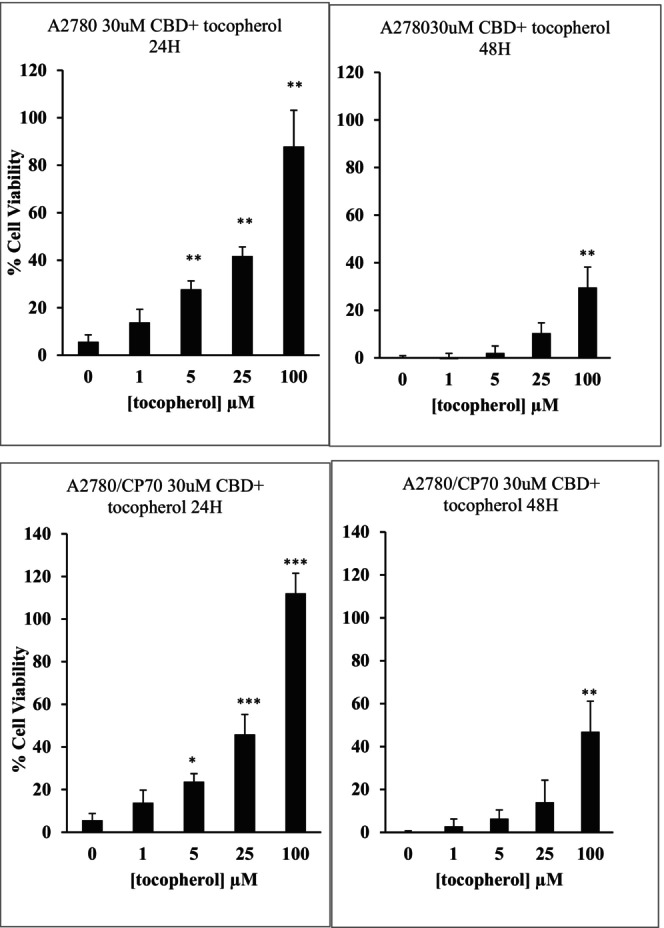
The cytotoxic effect of 30 μM of CBD alone and in combination with tocopherol (1 nM to 100 μM) on the A2780 and A2780/CP70 cell line after 24 and 48 h contact time. Data represent the mean ± SE of the mean of *n* = 4, *p < .05, **p < .01, and ***p < .001 compared to control values.

Pre‐treatment with α‐tocopherol reduced the cytotoxicity induced by CBD following 24 h contact time. For example, CBD alone at 30 μM induced approximately 90% cytotoxicity in cells after 24 h contact time. This value was significantly reduced in the presence of varying concentrations of α‐tocopherol when tested on both carcinoma cells. However, the latter trend was not observed when cells were treated for 48 h (Figure 9). Preliminary experiments showed that α‐tocopherol when used alone up to 100 μM did not compromise the viability of the cells.

### 3.8 Cannabinoid receptor involvement in mediating cytotoxicity to CBD and CBG

**FIGURE 10:**
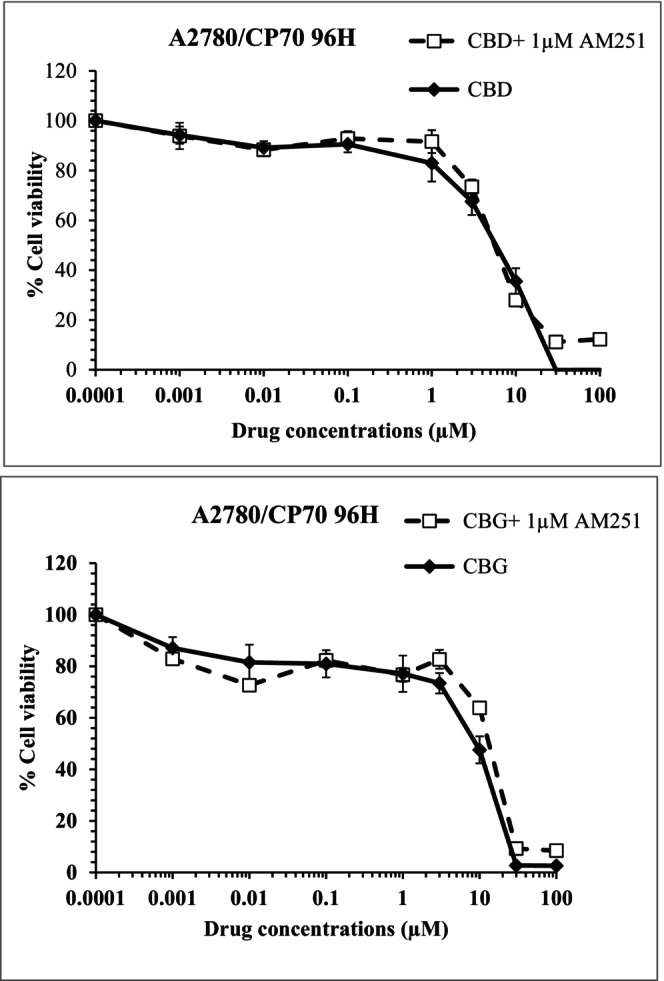
Representative data showing the effect of CBD or CBG alone and in the presence of 1 μM of AM251 over the exposure time of 96 h. Data represent the mean ± SE of the mean *n* = 3.

Pre‐treatment with AM251 and AM630 alone, CB_1_ and CB_2_ receptor antagonists, respectively, induced cytotoxicity at concentrations higher than 1 μM in both carcinoma cells. The cytotoxicity induced by CBD and CBG (1 nM–100 μM) were not modified in the presence of a sublethal dose of 1 μM AM251 in A2780 cells (results not shown). However, in the presence of AM251, when CBG was tested on A2780/CP70 cells, the IC_50_ was significantly (*p* < .05) higher (13.4 ± 0.7 μM) than that in the absence of the antagonist (7.1 ± 1.2 μM; Figure 10).

## 4 DISCUSSION

In the present study, CBD and CBG induced time‐ and concentration‐dependent cytotoxicity in cisplatin‐sensitive and ‐resistance ovarian carcinoma cells. When compared to non‐cancer cells, both cannabinoids demonstrated high selectivity in inducing cytotoxicity. Pre‐treatment with CBD over 96 h contact time induced a stronger cytotoxicity with IC_50_ values of 3.5 and 5.8 μM compared to CBG with IC_50_ values of 5.7 and 7.1 μM in A2780 and A2780/CP70 carcinoma cells, respectively. Furthermore, the exposure to CBD and CBG for 24 h induced significant cytotoxicity when cannabinoids were withdrawn from the cells.

CBD induced comparable cytotoxicity to carboplatin with an IC_50_ of 2.6 μM in A2780 cells, and a much higher cytotoxicity was induced by both cannabinoids in resistant carcinoma cells compared to when carboplatin used alone in A2780/CP70 cells. Furthermore, cannabinoids when used at a sublethal concentration in combination with carboplatin induced a selective synergistic effect in both carcinoma cell lines compared to non‐carcinoma cells. The ovarian cancer is associated with a diversity of mutations and histological types, it is difficult to get the desired therapeutic effect with a single drug.^12^, ^37^, ^38^ Previous studies have also suggested the potential of cannabinoids in clinical therapies.^39^ CBD induced a synergistic effect when it was combined with bortezomib, a protease inhibitor, on multiple myeloma cells and also in breast cancer.^40^, ^41^ The increase in the efficacy of carboplatin when applied in combination with cannabinoids may be due to the inhibitory effects of the cannabinoids on ABCC1 receptors. Previous studies showed that plant cannabinoids inhibited ABCC1 in ovarian cancer cells, and CBD exhibited the highest inhibition effects. ABCC1 is a multidrug resistance transportation receptor (MRP1), which involves in the efflux of anticancer drugs in phase II metabolism (Holland et al., 2008).^42^ By inhibiting ABCC1 receptor, cannabinoids may block the efflux of carboplatin leading to an enhanced efficacy on ovarian cancer cells. However, this could be determined by directly measuring the intracellular levels of carboplatin when combined with CBD or CBG using mass spectrometry. The synergistic effects may be due to multiple mechanisms of action and certainly a combination of drugs that exert different or nonoverlapping mechanisms of action could improve the therapeutic effect.^38^, ^43^ Further experiments are required to substantiate the involvement of ABCC1 receptors in mediating cytotoxicity induced by CBD and CBG. Nevertheless, the results suggests that the dose of chemotherapeutic drugs which patients receive can be reduced when used in combination with cannabinoids without the loss of activity.

To investigate whether changes in the viability of the cells induced by cannabinoids can lead to cell death via apoptosis, further experiments were designed to investigate the mitochondrial membrane potential changes induced by cannabinoids.^44^ Both CBD and CBG induced a loss in membrane potential in A2780 and A2780/CP70 cells in a dose‐dependent manner suggesting the induction of apoptosis in both cell lines. CBD was shown to induce apoptosis at lower concentrations compared to CBG on both ovarian cancer cells, which correlated with the IC_50_ values demonstrated earlier. Previous studies have also reported the induction of apoptosis by CBD in breast, prostate, glioma, and colon cancers, whereas CBG‐induced apoptosis was observed in prostate and colon cancers.^15^, ^29^, ^45^, ^46^

The induction of apoptosis by CBD and CBG in ovarian cancer cells was further confirmed by quantifying the Annexin V binding to extracellular exposed phosphatidylserine.^47^ The results suggested that cannabinoids induced cytotoxicity by affecting Annexin V pathway. Moreover, further experiments showed that the apoptosis induced by cannabinoids was also caspase 3/7 enzyme dependent. There are several different mechanisms of apoptosis, including the extrinsic and intrinsic apoptosis pathways, which are both caspase‐dependent and caspase‐independent mechanisms.^48^ In line with our studies, CBD was shown to induce caspase‐dependent apoptosis in various cancers such as breast, prostate, leukemia, while CBG has been shown to exert its cytotoxicity in prostate cancer cells by induction of the intrinsic apoptotic pathway.^46^, ^49^ The involvement of caspases 8 and 9 remains to be investigated.

In the present study, the attenuation of the cytotoxic effects of cannabinoids in the presence of an antioxidant, α‐tocopherol suggested the induction of ROS in both carcinoma cells. Mitochondrial dysfunction is one of the main reasons for the production of reactive oxygen species. Previous studies suggested the production of ROS following treatment with CBD in glioma cells.^50^ Then later studies also reported similar observations when cannabinoids were used in glioma as well as other carcinoma cells such as breast and colon cancer cells.^29^, ^46^, ^51^, ^52^ The accumulated studies indicated that CBD could activate apoptosis through the mitochondrial pathway and induce cell damage due to an increase in the level of ROS. This increase leads to the reduction of mitochondrial membrane potential, which subsequently increases the release of cytochrome C into the cell cytoplasm leading to apoptosis.^53^, ^54^ Therefore, it may be suggested that in ovarian cancer cannabinoids also induce cellular processes involving ROS signaling, which may be due to an impaired mitochondrial function.

In the present study the cell cycle arrest was induced by cannabinoids at G0–G1 phase. In the cell division process, cell cycle arrest can occur as a result of DNA damage or errors which cannot be repaired.^55^ The damage to the DNA will activate ATM/ATR signaling which involves p53 and consequently an increase in the level of p21. ATM/ATR signaling mediates G0–G1 arrest by regulating the expression of p53.^56^ Activation of ATM during DNA damage increases the expression of p21 and downregulates the expression of p53 protein. In addition, during the cell cycle, the cyclin‐dependent kinase inhibitor, p27 is activated and then binds to cyclin D, which subsequently inhibits the catalytic activity of Cdk4. The later will prevent the phosphorylation of retinoblastoma proteins leading to the prevention of pRb from releasing transcription factors which are essential for the progression of cells through the G1 and S phase.^57^ In the present study, the increase in G0/G1 correlated with a decrease in S phase when A2780 cells were treated with CBD. This may indicate the possibility of G1 growth arrest. It is possible that cannabinoids increase the level of ROS which leads to DNA damage. The damage to the DNA is detected by ATM/ATR signal, which then causes upregulation of p21 and downregulation of p53 as well as affecting p27, leading to cell arrest at the G0–G1 phase. The latter event is in addition to the loss of mitochondrial membrane potential, which may also lead to an increase in the level of ROS.^53^, ^58^

The cell cycle analysis of A2780 cells treated with CBG at 50 μM demonstrated the possible growth arrest at S phase, while in A2780/CP70 cells increased doses of CBG induced growth arrest in the S phase and G2/M phase. Nevertheless, the results clearly show that apoptotic cell death is also likely to be linked to alterations in the cell cycle program and more importantly, cannabinoids induce differential effects in carcinoma cells depending on their sensitivity to chemotherapeutic drugs.

AM251 is known as a CB_1_ receptor antagonist/inverse agonist with some non‐CB receptor‐dependent activity including anti‐tumor activity against pancreatic carcinoma cells.^59^, ^60^ In the present study, in line with previous studies, AM251 when used alone induced cytotoxicity at concentrations higher than 1 μM. In addition, the cytotoxicity induced by CBD or CBG when tested in the presence of AM251, which itself was used at a concentration which is known to antagonize CB_1_ receptors and nonlethal dose, was not modified in A2780 carcinoma cells. However, the cytotoxicity induced by CBG was significantly reduced in the presence of AM251 in cisplatin‐resistant cells, A2780/CP70 cells. This suggests that the CBG‐induced cytotoxicity in A2780/CP70 cells is at least partly mediated via CB_1_ receptors. Further experiments using knockdown cells are required to further substantiate the data.

The application of AM630 alone, a CB2 receptor antagonist/inverse agonist,^61^ induced cytotoxicity at concentrations higher than 1 μM. However, when AM360 was applied prior to the addition of CBD or CBG, it failed to modify the cytotoxicity induced by CBD and CBG. In line with other studies, the results suggested the unlikely involvement of CB_2_ receptors in mediating cytotoxicity induced by both cannabinoids in ovarian carcinoma cells.^15^

Although the present study investigated several pathways involved in apoptosis in mediating the cytotoxicity induced by cannabinoids, however, it is worth indicating that one of the limitations of this work is that CBD and CBG have been screened against a limited number of human ovarian cancer cells. CBD and CBG encouragingly showed similar activity toward the A2780/CP70 cisplatin‐resistant line compared to the parental A2780 cell line indicating that they can overcome platinum‐based cross‐resistance, however, future studies should include additional ovarian cancer cell lines with genetic variations. Another limitation is that the non‐cancer cells are not tissue matched, nevertheless the results provided some indication of selectivity prior to any possible progression towards in vivo studies. Further future experiments could also be carried out to investigate cannabinoid receptor expression levels in cannabinoid treated and non‐treated cells. While the present study suggested the involvement of Annexin V, mitochondrial membrane potential change and a cell cycle arrest in mediating apoptosis in cannabinoids‐induced cytotoxicity, future experiments can be designed to investigate the level of expression of some key proteins such as p53, Bax, and Bcl‐2 to further substantiate the mechanism of action of cannabinoids. With the recent availability of CRISPER‐CAS 9 technology future experiments should focus on investigating the exact site of action of cannabinoids.

## 5 CONCLUSION

Cannabinoids induced selective and significant cytotoxicity in ovarian cisplatin‐sensitive and ‐resistant carcinoma cells. CBD was shown to have higher potency compared to CBG. Both CBD and CBG induced synergistic effects when combined with carboplatin at sublethal dose of 100 nM. CBD in combination with carboplatin showed the strongest synergistic effect, and the synergism was ovarian cancer selective. The cytotoxicity induced by cannabinoids is likely to be through apoptotic mechanisms affecting mitochondrial membrane potential loss, Annexin V and caspase 3/7 activity, as well as cell cycle arrest. Furthermore, the cytotoxicity is likely to involve ROS as it was ameliorated in the presence of an antioxidant. The present study also highlighted the likely involvement of CB_1_ receptors in mediating CBG‐induced cytotoxicity in platin‐resistant cells, and further experiments are required to substantiate the results. It should be noted that the application of CBD in clinical settings did not induce significant side effects or detrimental effects on physiological parameters (heart rate, blood pressure, and body temperature) and psychological functions. Furthermore, high doses such as 1500 mg/day of CBD were well tolerated in humans.^62^, ^63^ With the selective cytotoxicity and the synergistic effects of cannabinoids observed in the present study when combined with carboplatin, clinical studies are encouraged to validate the effectiveness of combination therapies for the treatment of ovarian cancer particularly in patients resistant to chemo drugs. Indeed, multiple mechanisms of action activated by cannabinoids in inducing cytotoxicity in tumor cells would be beneficial for counteracting the resistance acquired in a specific pathway with the use of current cancer medications. With the recent data supporting the tolerability and safety profile of cannabinoids in clinical studies, the present results suggest that cannabinoids are potential candidates for combination therapy strategies.

## AUTHOR CONTRIBUTIONS

Kartheek K. Y. Sooda, obtaining the results for the PhD program. Simon J. Allison, supervision of the PhD, reviewing the manuscript. Farideh A. Javid, supervision of the PhD, writing, reviewing, and editing the manuscript.

## CONFLICT OF INTEREST STATEMENT

The authors have no conflict of interest.

## ETHICS STATEMENT

Not applicable.

## Data Availability

Data can be made available when the paper is published.
